# Pyrolysis temperature shapes biochar-mediated soil microbial communities and carbon-nitrogen metabolism

**DOI:** 10.3389/fmicb.2025.1657149

**Published:** 2025-09-26

**Authors:** Guihong Ren, Wentao Shi, Wenwen Li, Jinlong Wang, Chunjuan Wang, Guiyun Zhao

**Affiliations:** ^1^College of Science, Beihua University, Jilin, China; ^2^Traditional Chinese Medicine Biotechnology Innovation Center in Jilin Province, Beihua University, Jilin, China

**Keywords:** *Flammulina velutipes* residue, biochar, pyrolysis temperature, soil microbial community, seedling growth, carbon and nitrogen metabolism

## Abstract

**Introduction:**

Biochar derived from agricultural residues has potential to improve soil quality and regulate microbial communities, but its effect depends strongly on pyrolysis temperature.

**Methods:**

In this study, biochar prepared from Flammulina velutipes residue at 200 °C, 300 °C, and 400 °C was applied to cucumber seedling cultivation to evaluate its influence on soil physicochemical properties, microbial community structure, and functional metabolism.

**Results:**

Results showed that soil pH increased significantly with biochar addition, from 5.00 in the control to 6.17 at 400 °C, while soil organic matter reached the highest level in the 400 °C treatment (90.03 g·kg^−1^). Available phosphorus and potassium were also enhanced, with maximum values of 731.81 mg·kg^−1^ and 481.68 mg·kg^−1^, respectively. Seedling growth responded differently to pyrolysis temperatures: the 300 °C biochar treatment increased above-ground biomass to 0.18 g and total biomass to 0.214 g per plant, significantly higher than the control (0.124 g). Metagenomic sequencing revealed shifts in dominant microbial phyla, with Acidobacteriota enriched at higher temperatures, and alpha diversity indices (Chao1, ACE, Sobs) increased under 400 °C biochar. Functional analysis indicated that carbon metabolic genes (e.g., acetyl-CoA synthesis, TCA cycle) were optimized at moderate to high temperatures, whereas nitrogen metabolism showed divergent responses, with nitrate reduction favored at 300 °C and nitrite reduction at 400 °C. Regression analysis demonstrated a positive correlation between microbial diversity and carbon metabolism genes (*R*^2^ = 0.75), but a negative correlation with nitrogen metabolism genes (*R*^2^ = 0.56). Redundancy analysis further identified ammonium nitrogen, acid phosphatase, and catalase as key drivers of microbial community and functional gene structure.

**Discussion:**

Overall, these findings highlight that biochar from mushroom residue, particularly produced at 300–400 °C, improves soil fertility, regulates microbial community composition, and modulates carbon and nitrogen metabolic processes, thereby enhancing cucumber seedling growth.

## 1 Introduction

Soil degradation, nutrient depletion, and declining soil organic matter content have become major constraints to sustainable agriculture worldwide (Chaplot, 2021; Matisic et al., 2024). To address these challenges, biochar has gained increasing attention as a soil amendment due to its ability to improve soil physicochemical properties, enhance microbial activity, and promote plant growth (Murtaza et al., 2021; Yu et al., 2019). Biochar, a carbonaceous material derived from the pyrolysis of organic biomass under oxygen-limited conditions, has been widely studied for its role in increasing soil carbon sequestration, regulating soil pH, and influencing microbial-driven nutrient cycling (Lehmann and Joseph, 2024; Sheng and Zhu, 2018). However, the effects of biochar on soil quality and plant performance largely depend on its physicochemical properties, which are determined by feedstock type and pyrolysis temperature (Ippolito et al., 2020; Tomczyk et al., 2020).

Among various biochar feedstocks, agricultural residues from mushroom cultivation offer a promising and sustainable source for biochar production. *Flammulina velutipes*, commonly known as enoki mushroom, is widely cultivated, generating substantial amounts of organic waste rich in lignocellulose and other nutrients (Rezaeian and Pourianfar, 2017). Transforming *Flammulina velutipes* residue into biochar via pyrolysis could provide an effective waste management strategy while enhancing soil fertility and microbial activity. However, limited studies have investigated how the pyrolysis temperature of *Flammulina velutipes* residue biochar affects soil properties, microbial communities, and plant growth. Understanding these effects is essential for optimizing biochar application in agricultural systems.

Pyrolysis temperature is a key determinant of biochar properties, influencing its surface area, pore structure, pH, nutrient content, and carbon stability (Ippolito et al., 2020; Zhang et al., 2022). Biochar produced at lower temperatures (200–300 °C) tends to retain more labile organic compounds and has a greater ability to support microbial activity, while high-temperature biochar (≥400 °C) is more recalcitrant, providing long-term carbon storage but with reduced nutrient availability (Tomczyk et al., 2020). Recent studies have shown that biochar application can alter soil nitrogen cycling, phosphorus availability, and potassium retention, depending on the pyrolysis conditions and biochar composition (Gao et al., 2019; Yu et al., 2018). However, the specific mechanisms by which biochar at different temperatures affects soil nutrient dynamics and microbial metabolism remain insufficiently understood.

Microbial communities are highly sensitive to changes in soil physicochemical properties, and biochar can significantly reshape microbial diversity, composition, and functional potential (Qiu et al., 2022; Zhu et al., 2022). While some studies suggest that biochar enhances microbial diversity by providing habitat niches and altering nutrient availability, others indicate that high-temperature biochar may suppress microbial activity due to its lower bioavailable carbon content (Lan et al., 2018; Liao et al., 2022). Furthermore, the impact of pyrolysis temperature on functional microbial processes such as carbon and nitrogen metabolism remains unclear, particularly in biochar-amended soils used for seedling cultivation.

*Flammulina velutipes* (commonly known as enoki mushroom) is one of the most widely cultivated edible fungi worldwide, with considerable nutritional and economic value (Sharma et al., 2021). It represents an ideal experimental organism due to its rapid growth cycle, relatively simple cultivation requirements, and high sensitivity to environmental and substrate changes (Xie et al., 2017). These characteristics make *F. velutipes* particularly suitable for assessing the ecological effects of biochar amendments, as its growth and metabolic activities can provide rapid and measurable responses to changes in substrate properties and microbial community dynamics. Moreover, *F. velutipes* secretes a broad spectrum of extracellular enzymes that play an essential role in lignocellulose degradation and nutrient cycling (Liu et al., 2022), which allows for a more precise evaluation of how biochar influences microbial functional potential, especially those related to carbon and nitrogen metabolism. Compared with other fungi or plant-based systems, *F. velutipes* cultivation offers a highly standardized and reproducible experimental platform, thereby minimizing the interference of uncontrolled environmental factors and enabling clearer mechanistic insights. By incorporating *F. velutipes* into our experimental design, this study not only addresses the general effects of biochar on microbial community composition but also captures the fungus-specific interactions that are of practical significance for edible mushroom production and sustainable agricultural management.

Given the knowledge gaps in biochar research, this study aims to investigate the influence of *Flammulina velutipes* residue biochar produced at different pyrolysis temperatures on soil properties, microbial communities, metabolic pathways related to C and N cycling., and plant growth during seedling cultivation. The specific objectives of this study are: I. To evaluate the effects of different pyrolysis temperatures on soil pH, nutrient availability, and organic matter content. II. To assess how biochar influences cucumber (*Cucumis sativus*) seedling growth and biomass accumulation. III. To assess the impact of biochar amendments on soil microbial diversity, community composition, and functional gene expression associated with carbon and nitrogen cycling. IV. To identify the environmental factors driving microbial community structure and functional gene distributions.

We hypothesize that biochar produced at moderate pyrolysis temperatures (~300 °C) will optimize soil nutrient availability, enhance microbial diversity, and promote seedling growth by creating a balanced soil microenvironment. In contrast, biochar produced at lower temperatures (~200 °C) may favor microbial activity but provide less stable nutrient retention, whereas high-temperature biochar (~400 °C) may enhance soil pH and long-term carbon sequestration but reduce microbial diversity and functional activity. This study provides novel insights into the role of *Flammulina velutipes* residue biochar in soil microbial ecology and plant growth, contributing to the sustainable management of agricultural waste. By elucidating the mechanisms through which pyrolysis temperature influences soil biochemical processes and microbial functions, our findings will support the development of optimized biochar application strategies for improving soil fertility and crop productivity in sustainable agriculture.

## 2 Materials and methods

### 2.1 Experimental materials

The *Flammulina velutipes* residue used in this experiment was obtained from Changchun Xueguo Gaorong Biotechnology Co., Ltd. To prepare biochar, fresh mushroom residue was pyrolyzed following the procedure outlined below. Initially, the mushroom residue was air-dried and then crushed. The particles with a size range of 0.2 to 2 mm were selected for the pyrolysis process. The residue was pyrolyzed in a muffle furnace at three different temperatures—200 °C, 300 °C, and 400 °C—under anoxic conditions for a duration of 30 min, resulting in *Flammulina velutipes* residue biochar.

The soil used for the seedling cultivation experiment was nutrient-rich soil, previously utilized for cucumber seedling cultivation. The basic physical and chemical properties of this soil were as follows: pH 6.77, soil organic matter content of 242.02 g·kg^−1^, available phosphorus of 1.01 g·kg^−1^, available potassium of 12.9 g·kg^−1^, and total nitrogen of 9.29 g·kg^−1^. Cucumber seedlings of the variety “Chunqiu Mici” were used in this study.

To maintain consistent soil moisture, all planting trays were placed in a controlled growth chamber, and soil water content was adjusted daily to approximately 60% of the water-holding capacity by weighing the trays and adding deionized water when necessary. The planting trays (length 40 cm × width 30 cm × height 10 cm) were made of polyethylene and were equipped with a drainage layer of fine gauze at the bottom to allow excess water to drain while preventing soil and biochar loss.

To avoid potential upward migration and loss of biochar during watering, we used a bottom-watering approach, in which water was supplied slowly and evenly to the soil surface with a fine mist sprayer to minimize water flow disturbance. Moreover, biochar was homogeneously mixed into the soil before transplantation, ensuring stable distribution across the soil profile throughout the experiment. No visible migration or loss of biochar was observed during the cultivation period.

### 2.2 Grouping of seedling cultivation experiment

The seedling cultivation experiment was organized into four treatment groups, including one control group and three experimental groups, each with different biochar treatments. The treatments were as follows: TEP1 (Control group): Basic soil with no biochar addition. TEP2: Basic soil amended with 4% (w/w) *Flammulina velutipes* residue biochar pyrolyzed at 200 °C. TEP3: Basic soil amended with 4% (w/w) *Flammulina velutipes* residue biochar pyrolyzed at 300 °C. TEP4: Basic soil amended with 4% (w/w) *Flammulina velutipes* residue biochar pyrolyzed at 400 °C. The biochar addition rate of 4% (w/w) was selected based on preliminary experiments showing improved cucumber seedling performance without adverse changes in soil density or salinity. This rate also falls within the typical range used in seedling cultivation studies—for example, 2.5% (w/w) in nursery experiments (Simiele et al., 2022) or 1.5% (w/w) for maize seedling growth trials (Ali et al., 2021). The experiment utilized 50-hole plug trays for the cultivation of cucumber seedlings. Two cucumber seeds were sown per hole, and one seedling was left in each hole after emergence. Standard water management practices were applied throughout the experiment. The cultivation environment was controlled, with a 16-h light period at 25 °C and an 8-h dark period at 20 °C.

### 2.3 Determination of indicators and methods

#### 2.3.1 Morphological indicators of cucumber seedlings

To assess the impact of biochar on cucumber seedling growth, the following morphological parameters were measured at 30 days after sowing: Plant Height: The distance from the root-stem junction to the base of the growth point was measured using a ruler (cm). Stem Diameter: The diameter at the point where the seedling stem meets the substrate was measured using a vernier caliper (mm). Biomass: The above-ground and underground parts of the seedlings were collected, killed at 105 °C for 20 min, and then dried at 80 °C to a constant weight. The biomass was weighed to determine the total dry weight. Leaf Area: The leaf surface area was imaged using a Canon Scan LIDE220 scanner, and the leaf area was calculated using Image software. Strong-Seedling Index: The quality of cucumber seedlings was determined using the strong-seedling index, calculated as: Strong-seedling index = (Stem diameter / Plant height + Underground dry weight / Aboveground dry weight) × Dry weight per plant.

#### 2.3.2 Physiological indicators of cucumber seedlings

Physiological parameters were assessed at 20 and 30 days after sowing (when the adult leaves had emerged). The following physiological indicators were measured: Root Activity: Root activity was measured using the TTC method, which assesses the dehydrogenase activity in roots. Superoxide Dismutase (SOD) Activity: The activity of SOD was measured using the nitroblue tetrazolium photoreduction method. Phenylalanine Ammonia-Lyase (PAL) Activity: PAL activity was measured using ultraviolet spectrophotometry, which quantifies the enzyme’s role in lignin biosynthesis.

#### 2.3.3 Soil enzyme activity

Soil enzyme activity was determined by sampling the substrates in the plug trays at 10, 20, and 30 days after sowing. The following soil enzymes were measured: Urease (URE): Urease activity was determined using sodium phenolate-sodium hypochlorite colorimetry. Invertase: Invertase activity was measured using 3,5-dinitrosalicylic acid colorimetry. Catalase (CAT): Catalase activity was quantified by ultraviolet spectrophotometry. Phosphatase: Phosphatase activity was determined using disodium phenyl phosphate colorimetry.

#### 2.3.4 Microbial community structure analysis

To investigate the effects of biochar treatments on soil microbial communities, metagenomic sequencing was conducted on soil samples collected 30 days after sowing. Total DNA was extracted from soil samples using the PowerSoil DNA Isolation Kit (MoBio Laboratories, Carlsbad, CA, USA), following the manufacturer’s protocol. DNA concentration and integrity were verified using a NanoDrop 2000 spectrophotometer and agarose gel electrophoresis.

Shotgun metagenomic sequencing was performed using the Illumina NovaSeq 6,000 platform (Illumina Inc., San Diego, CA, USA). Raw reads were quality-filtered using Trimmomatic to remove low-quality sequences and adapters. High-quality reads were assembled using MEGAHIT, and open reading frames (ORFs) were predicted using Prodigal. Functional annotation of predicted genes was performed against the KEGG (Kyoto Encyclopedia of Genes and Genomes), eggNOG, and CAZy databases using DIAMOND with an e-value cutoff of 1e−5.

For taxonomic profiling, metagenomic reads were aligned to the NCBI NR database using DIAMOND, and taxonomic classification was performed using MEGAN. Microbial community structure was analyzed at multiple taxonomic levels (phylum, class, order, family, and genus), and diversity indices including alpha diversity (Chao1, ACE, Shannon, Simpson) and beta diversity (Bray–Curtis dissimilarity) were calculated using the vegan package in R.

Non-metric multidimensional scaling (NMDS) was used to visualize differences in microbial community composition among treatments. Statistical significance of group separation was tested using permutational multivariate analysis of variance (PERMANOVA). Additionally, functional gene profiles were compared among treatments to assess the impact of pyrolysis temperature on microbial metabolic potential related to carbon and nitrogen cycling.

### 2.4 Statistical analysis

All statistical analyses were conducted using R software (R Core Team, Vienna, Austria). Data were first checked for normality and homogeneity of variance using the Shapiro–Wilk test (shapiro.test function in the stats package) and Levene’s test (leveneTest function in the car package). For comparisons among different pyrolysis temperature treatments (TEP1, TEP2, TEP3, and TEP4), a one-way analysis of variance (ANOVA) was performed using the aov function from the stats package. When significant differences were detected (*p* < 0.05), Tukey’s Honest Significant Difference (HSD) test was applied (TukeyHSD function in the stats package) to determine pairwise differences between treatments.

For soil microbial diversity analysis, alpha-diversity indices (Chao1, ACE, Shannon, and Simpson indices) were calculated using the vegan package (diversity function). The differences in microbial community composition were assessed using non-metric multidimensional scaling (NMDS) based on Bray–Curtis dissimilarity (metaMDS function in the vegan package). Statistical significance of beta-diversity differences among treatments was tested using permutational multivariate analysis of variance (PERMANOVA) (adonis2 function in the vegan package) (Anderson, 2001).

To identify the key environmental factors influencing microbial community structure and functional gene distribution, redundancy analysis (RDA) was conducted using the vegan package (rda function) (Oksanen et al., 2016). The significance of explanatory variables was determined by Monte Carlo permutation tests (anova.cca function in vegan). All visualizations, including boxplots, NMDS ordination plots, and RDA biplots, were generated using the ggplot2 and ggpubr packages for high-quality graphical representation.

## 3 Results

### 3.1 Soil properties and nutrient content

The application of *Flammulina velutipes* residue biochar at different pyrolysis temperatures significantly influenced soil properties (Table 1). The pH of the soil increased with biochar addition, with the highest pH observed in the TEP4 treatment (6.17 ± 0.125), which was significantly higher than that of the control group (TEP1: 5.0 ± 0.115) and those of the other biochar treatments (TEP2: 5.69 ± 0.083, TEP3: 5.7 ± 0.075). Electrical conductivity (EC) also increased, with the TEP3 treatment showing the highest EC (0.469 ± 0.036). The ammonium nitrogen (NH₄^+^-N) content was highest in TEP2 (141.29 ± 7.07 mg·kg^−1^), while nitrate nitrogen (NO_3_^−^-N) was significantly higher in TEP4 (190.15 ± 26 mg·kg^−1^) compared to other treatments. Available phosphorus (AP) and available potassium (AK) were significantly higher in TEP4, with AP reaching 731.81 ± 28.5 mg·kg^−1^ and AK at 481.68 ± 53.7 mg·kg^−1^. Soil organic matter (SOM) content was highest in TEP4 (90.03 ± 4.63 g·kg^−1^), with no significant difference compared to TEP3 (89.3 ± 2.97 g·kg^−1^). These findings indicate that biochar produced at higher pyrolysis temperatures (TEP3 and TEP4) significantly enhances soil pH, nutrient availability, and soil organic matter compared with low-temperature biochar.

**Table 1:** Effect of different pyrolysis temperatures of *Flammulina velutipes* residue biochar on soil properties.

Treatment	pH	EC	NH_4_^+^-N (mg·kg^−1^)	NO_3_^—^N (mg·kg^−1^)	Available phosphorus (AP, mg·kg^−1^)	Available potassium (AK, mg·kg^−1^)	Soil organic matter (SOM, g·kg^−1^)
TEP1	5 ± 0.115a	0.374 ± 0.022a	129.34 ± 12.7ab	171.9 ± 14.9ab	44.34 ± 7.07a	109.18 ± 4.37a	82.67 ± 1.83a
TEP2	5.69 ± 0.083b	0.385 ± 0.03a	141.29 ± 7.07a	149.02 ± 16.2ab	418.22 ± 42.6b	417.2 ± 53.7bc	83.76 ± 3.57a
TEP3	5.7 ± 0.075bc	0.469 ± 0.036b	136.33 ± 11.8ab	151.75 ± 11.6b	551.86 ± 61.9b	393.68 ± 58.5b	89.3 ± 2.97b
TEP4	6.17 ± 0.125d	0.459 ± 0.038b	127.08 ± 4.63b	190.15 ± 26a	731.81 ± 28.5b	481.68 ± 53.7c	90.03 ± 4.63b

Values are expressed as mean ± SD. Different lowercase letters in each column indicate significant differences between treatments (*p* < 0.05).

### 3.2 Seedling growth and biomass

Significant differences were observed in seedling growth and biomass across the pyrolysis temperature treatments (Table 2). The highest plant height (6.17 ± 0.423 cm) was observed in the TEP4 treatment, significantly greater than the TEP1 (3.76 ± 0.423 cm) and TEP2 (3.53 ± 0.776 cm) treatments. Similarly, the TEP3 group exhibited the highest above-ground biomass (0.18 ± 0.025 g), significantly higher than TEP1 (0.105 ± 0.016 g) and TEP2 (0.113 ± 0.018 g). Below-ground biomass was also highest in TEP3 (0.033 ± 0.007 g), while the lowest was found in TEP4 (0.015 ± 0.002 g). The total biomass followed a similar trend, with TEP3 having the highest total biomass (0.214 ± 0.026 g) compared to TEP1 (0.124 ± 0.018 g) and TEP2 (0.135 ± 0.011 g). These findings suggest that biochar produced at 300 °C (TEP3) enhanced seedling growth and biomass accumulation, particularly in above-ground and total biomass, while higher pyrolysis temperatures (TEP4) had a less pronounced effect on biomass production.

**Table 2:** Effect of different pyrolysis temperatures of *Flammulina velutipes* residue biochar on plant morphological indicators and biomass.

Treatment	Plant height (cm)	Stem diameter (cm)	Stem diameter/Plant height (cm)	Above-ground biomass (g)	Below-ground biomass (g)	Root to shoot ratio	Total biomass (g)	Zygotic Maturity Index
TEP1	3.76 ± 0.423a	2.34 ± 0.194a	0.633 ± 0.093	0.105 ± 0.016a	0.018 ± 0.002a	0.17 ± 0.036a	0.124 ± 0.018a	0.1 ± 0.021a
TEP2	3.53 ± 0.776ab	2.1 ± 0.213a	0.62 ± 0.109	0.113 ± 0.018a	0.018 ± 0.003a	0.17 ± 0.036a	0.135 ± 0.011a	0.106 ± 0.013a
TEP3	4.89 ± 0.405c	2.52 ± 0.223ab	0.512 ± 0.18	0.18 ± 0.025b	0.033 ± 0.007b	0.19 ± 0.048a	0.214 ± 0.026a	0.148 ± 0.015b
TEP4	6.17 ± 0.423c	2.73 ± 0.194b	0.45 ± 0.093	0.14 ± 0.016c	0.015 ± 0.002c	0.11 ± 0.036b	0.155 ± 0.018b	0.086 ± 0.021a

Values are expressed as mean ± SD. Different lowercase letters in each column indicate significant differences between treatments (*p* < 0.05).

### 3.3 Soil microbial community composition

**Figure 1:**
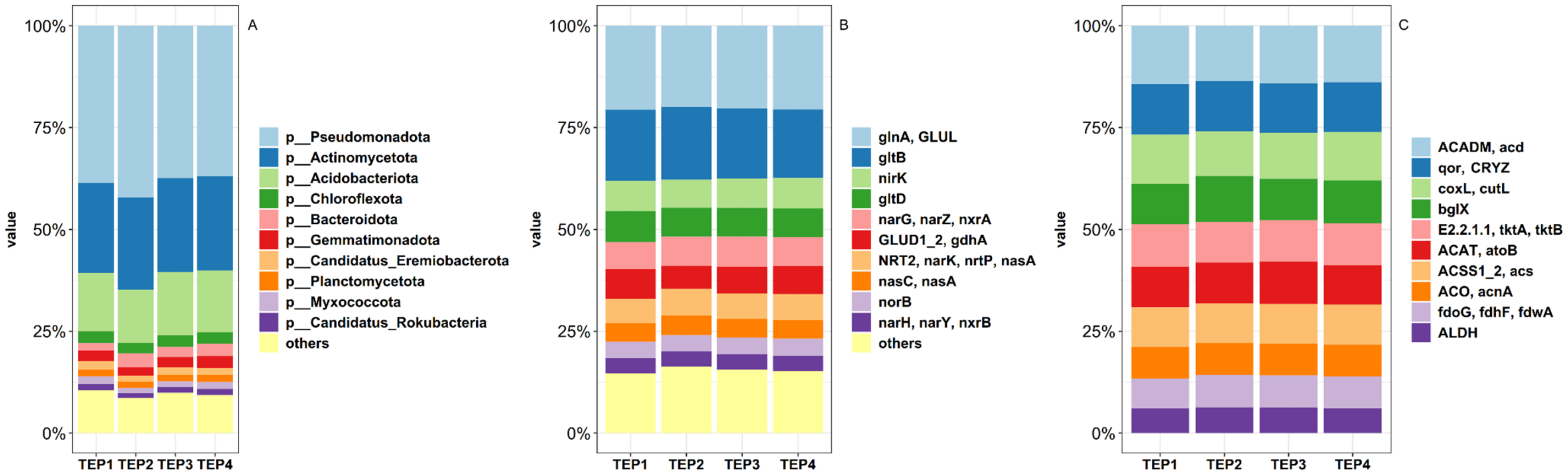
Comparison of soil microbial community composition **(A)** and distributions of and nitrogen **(B)** carbon **(C)** metabolism-related genes under different pyrolysis temperatures of *Flammulina velutipes* residue biochar.

Metagenomic sequencing revealed significant differences in the soil microbial community composition across the various pyrolysis temperature treatments. A total of 4 domains, 13 kingdoms, 237 phyla, 439 classes, 806 orders, 1,595 families, 5,502 genera, and 38,331 species were identified. At the phylum level, *Pseudomonadota*, *Actinomycetota*, *Acidobacteriota*, and *Chloroflexota* were the dominant groups (Figure 1A). *Pseudomonadota* showed significantly higher relative abundance in the TEP2 group compared to the TEP3 and TEP4 groups (*p* < 0.05), indicating that lower pyrolysis temperatures may favor these bacteria. In contrast, *Acidobacteriota* was more abundant in the TEP3 and TEP4 groups than in TEP2 (*p* < 0.05), suggesting that higher pyrolysis temperatures create conditions more suitable for their growth. *Chloroflexota* and *Gemmatimonadota* were significantly more abundant in the TEP2, TEP3, and TEP4 groups compared to TEP1 (*p* < 0.05), while no significant differences were observed in the relative abundance of *Actinomycetota* across treatments (Supplementary Figure S1). These results suggest that biochar, regardless of pyrolysis temperature, enhances the presence of certain phyla involved in organic matter degradation and nutrient cycling.

### 3.4 Influence of pyrolysis temperature on soil microorganisms and functions

**Figure 2:**
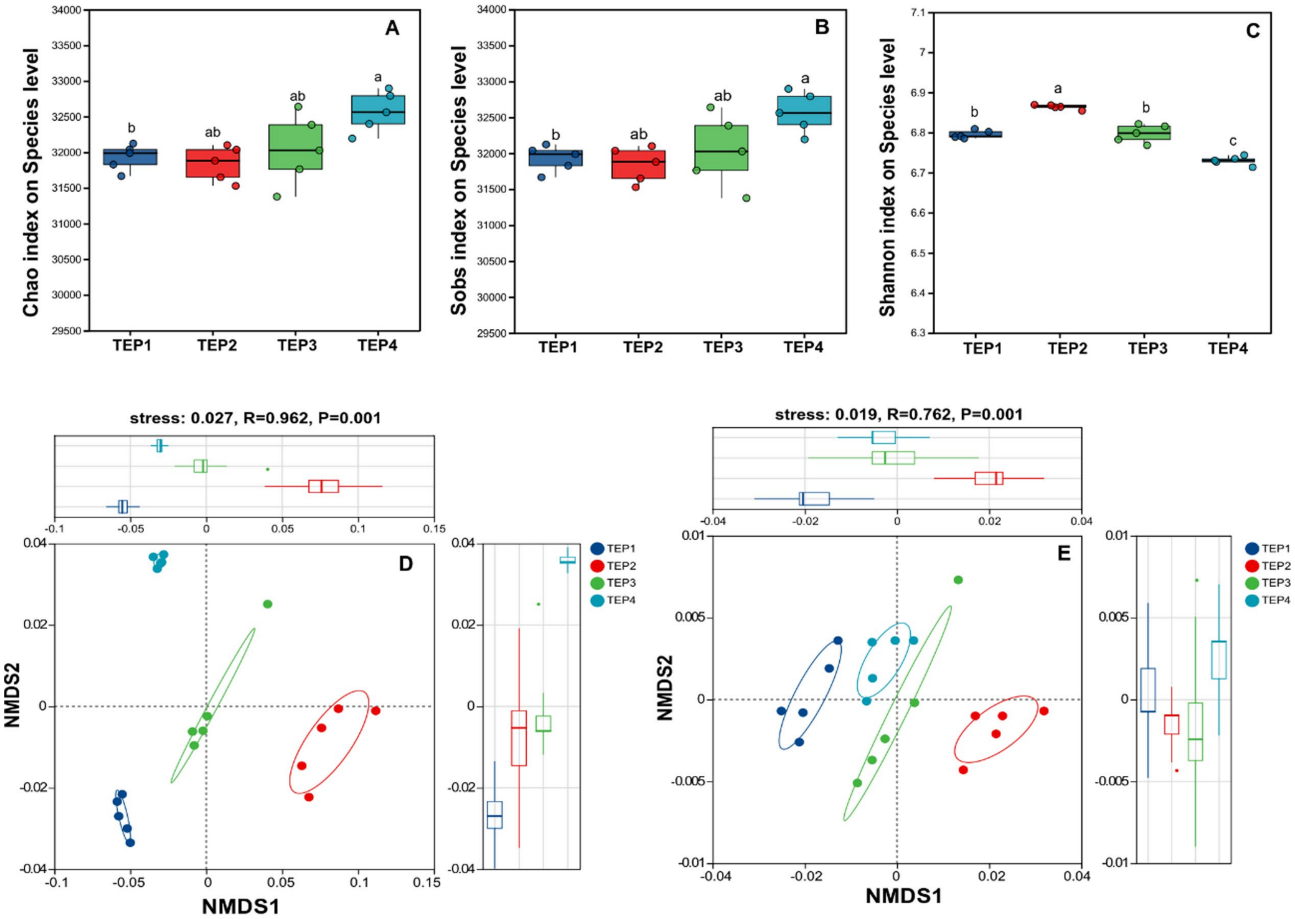
Microbial community and functional gene diversity analysis, **(A–C)** illustrate microbial diversity indices (Chao, Sobs, and Shannon, respectively) at the species level, comparing variations across TEP1–TEP4 treatments. **(D,E)** Present non-metric multidimensional scaling (NMDS) analyses, with **(D)** depicting microbial *β*-diversity (stress = 0.027, *R* = 0.962, *p* < 0.001) and **(E)** showcasing functional gene diversity (stress = 0.019, *R* = 0.762, *p* < 0.001), highlighting distinct clustering patterns among TEP1–TEP4 samples.

The analysis of *α*-diversity revealed a general increasing trend in the Chao, ACE, and Sobs indices with higher pyrolysis temperatures (Figure 2). Notably, the TEP4 group exhibited significantly higher indices compared to TEP1 and TEP2. In contrast, the Shannon index was highest in the TEP2 group, significantly surpassing the values in TEP1, TEP3, and TEP4 groups, indicating distinct differences in microbial diversity at varying temperatures. *β*-diversity analysis, using NMDS, clearly separated the microbial communities across the TEP1, TEP2, TEP3, and TEP4 groups, with a stress value of 0.027, *R* = 0.962, and *p* = 0.001. This separation was also evident in functional gene compositions, KEGG (stress: 0.019, *R* = 0.762, *p* = 0.001) analyses revealed significant differentiation in functional gene groups according to pyrolysis temperature treatments.

### 3.5 Influence of pyrolysis temperature on nitrogen metabolic processes and carbon metabolic processes

The analysis of carbon (C) metabolism revealed that pyrolysis temperature significantly influences key enzymatic activities (Figure 1B). Lower temperatures (TEP1) favored fatty acid degradation, as indicated by higher expression of ACADM acd, while moderate temperatures (TEP3 and TEP4) stabilized pathways like acetyl-CoA synthesis and the TCA cycle (ACSS1_2 acs, ACO acnA). Formate metabolism (fdoG fdhF fdwA) peaked at intermediate temperatures (TEP2) (Supplementary Figure S2), suggesting temperature-dependent regulation of specific C metabolic pathways. For nitrogen (N) metabolism (Figure 1C), extreme temperatures (TEP1 and TEP4) enhanced glutamine synthesis (glnA GLUL), while moderate temperatures (TEP3) optimized nitrate reduction (narG narZ nxrA). Nitrite reduction (nirK) was highest at TEP4, indicating a preference for higher pyrolysis temperatures. However, ammonia assimilation (GLUD1_2 gdhA) was suppressed at intermediate temperatures (TEP2) (Supplementary Figure S3). Overall, pyrolysis temperature plays a critical role in modulating both C and N metabolic processes, with distinct temperature ranges favoring specific enzymatic activities.

### 3.6 Regression analysis of the relationships between microbial diversity and carbon/nitrogen metabolism-related genes

**Figure 3:**
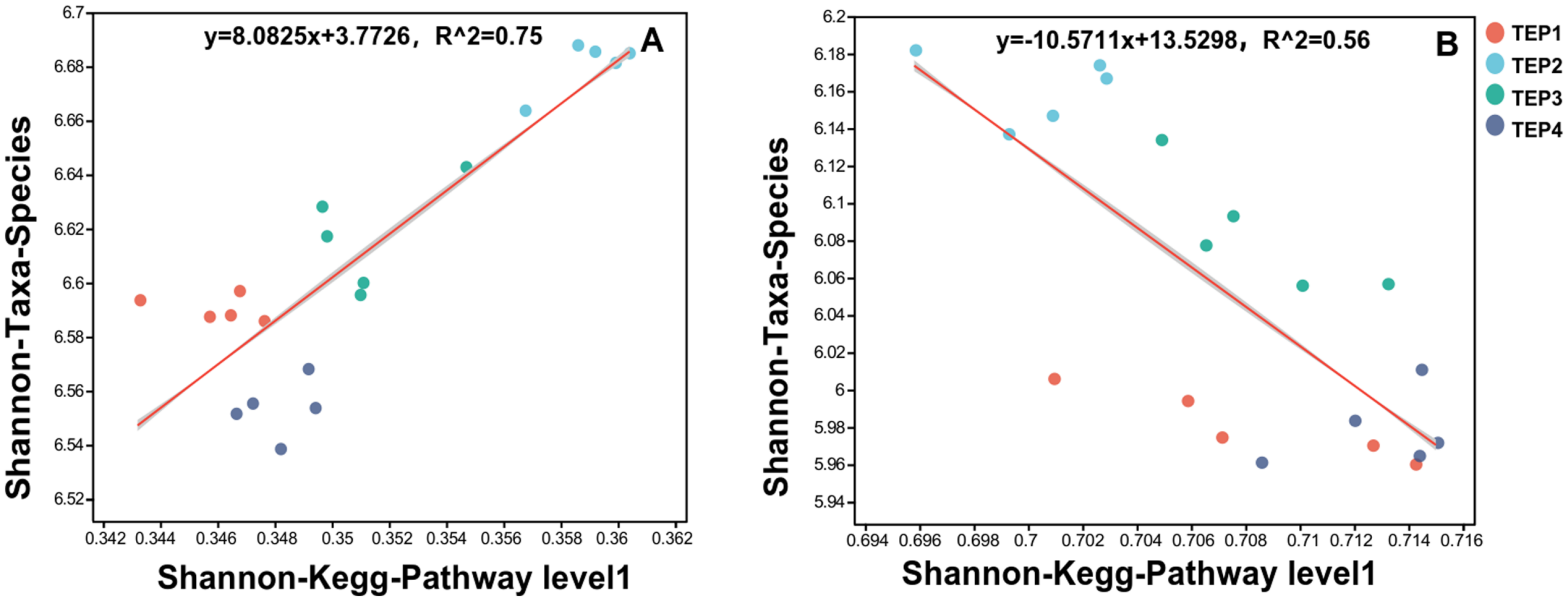
Regression analysis of the relationships between microbial diversity and carbon/nitrogen metabolism-related genes. **(A)** Regression analysis of the relationship between microbial diversity and carbon—metabolism-related genes. **(B)** Regression analysis of the relationship between microbial diversity and nitrogen—metabolism-related genes.

The regression analyses of microbial diversity and metabolic functions revealed contrasting relationships between carbon- and nitrogen-metabolism-related genes. A significant positive correlation was observed between microbial diversity and carbon metabolism-related genes (regression equation: y = 8.0825x + 3.7726, *R*^2^ = 0.75), indicating that increased carbon metabolic functional diversity promotes microbial species diversity. This suggests that enhanced carbon utilization pathways may support a wider range of microbial taxa by providing diverse energy sources and structural components. In contrast, nitrogen metabolism-related genes exhibited a negative correlation with microbial diversity (regression equation: y = −10.5711x + 13.5298, *R*^2^ = 0.56), suggesting that an increase in nitrogen-metabolic functional diversity may lead to a decline in microbial species diversity. This could be attributed to competitive exclusion among nitrogen-transforming microbial populations, where dominant species outcompete others under nitrogen-enriched conditions (Figure 3).

### 3.7 Environmental drivers of microbial community and functional gene structure: insights from RDA analysis

The RDA analysis revealed that NH4^+^-N (ammonium nitrogen) and SAPA (acid phosphatase activity) were key drivers of microbial community structure, explaining significant variance (*p* < 0.05). SCC (catalase activity) also played a crucial role, highlighting the importance of oxidative stress response. Additionally, PPAL (phenylalanine ammonia-lyase) and PCIIb (chlorophyll b) strongly influenced microbial composition, linking secondary metabolism and photosynthetic activity to community dynamics.

**Figure 4:**
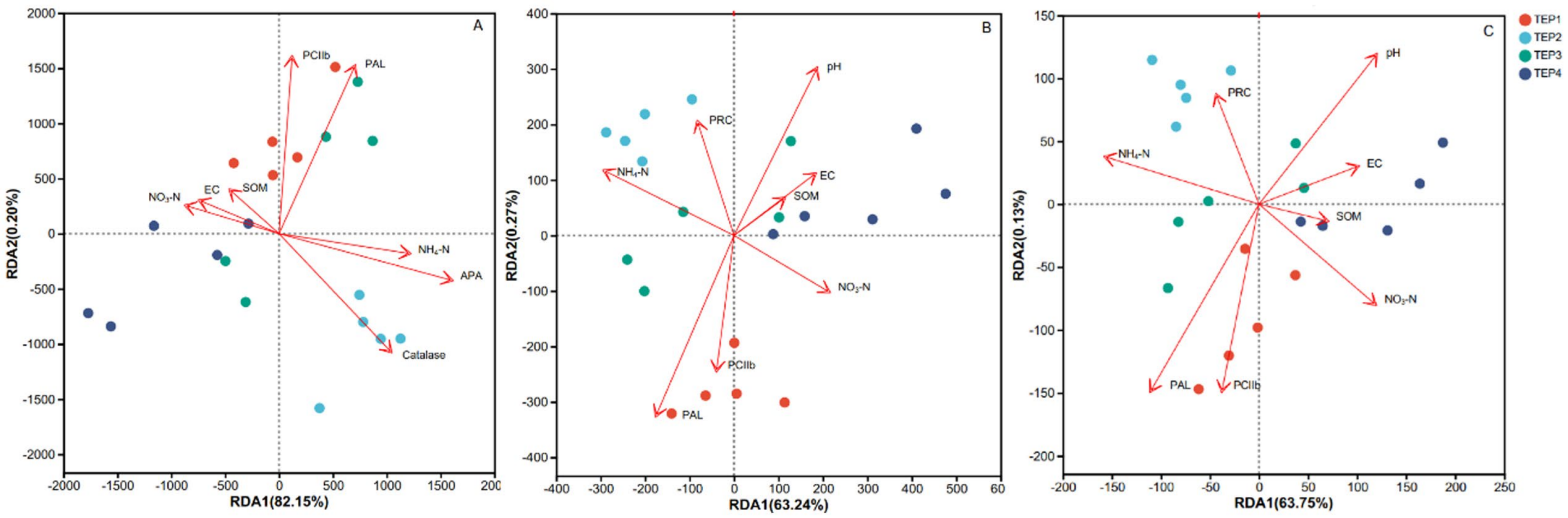
Redundancy analysis (RDA) of environmental factors influencing microbial communities and functional genes across treatments **(A–C)** illustrate RDA results for microbial communities and functional genes under different treatments (TEP1–TEP4). Colored points represent different treatments: TEP1 (red), TEP2 (light blue), TEP3 (green), and TEP4 (cyan). **(A)** RDA of microbial species, with arrows indicating key environmental factors such as EC (electrical conductivity), NO_3_^−^-N, NH_4_^+^-N, alkaline phosphatase activity (APA), polyphenol oxidase activity (PPO), phenylalanine ammonia-lyase (PAL), and catalase. **(B)** Presents the RDA of carbon metabolism-related genes, showing associations with factors such as pH, EC, soil organic matter (SOM), NO_3_^−^-N, and NH_4_^+^-N. **(C)** The RDA of nitrogen metabolism-related genes, with arrows pointing to factors including pH, EC, SOM, NO_3_^−^-N, NH_4_^+^-N, and key functional genes. The percentages in parentheses represent the proportion of variance explained by the respective RDA axes.

For KEGG functional gene structure, PPAL and PCIIb again emerged as significant factors, alongside pH, which showed a strong correlation (*p* = 0.011). These findings suggest that secondary metabolite biosynthesis and photosynthetic processes are critical in shaping functional gene diversity (Figure 4).

It should be noted that although the arrow length in the RDA biplot indicates the relative contribution of explanatory variables, it does not directly provide statistical strength. Therefore, we performed additional permutation tests, which confirmed that NH4^+^-N, SAPA, PPAL, PCIIb, and pH were significant drivers (*p* < 0.05), whereas EC and SOM had no significant impact. This further emphasizes the selective influence of specific environmental variables on microbial functions during biochar-amended seedling cultivation.

## 4 Discussion

The application of *Flammulina velutipes* residue biochar at different pyrolysis temperatures significantly influenced soil properties, microbial community composition, and metabolic functions, ultimately affecting seedling growth and biomass accumulation. Our findings demonstrate that pyrolysis temperature plays a critical role in modulating soil nutrient availability, microbial diversity, and functional gene expression, which are key drivers of plant growth and soil health.

Biochar amendments significantly influenced soil physicochemical properties, with higher pyrolysis temperatures increasing soil pH, available phosphorus (AP), and available potassium (AK). The increase in soil pH observed in the TEP4 treatment aligns with previous studies showing that high-temperature biochars exhibit stronger liming effects due to the removal of acidic functional groups and the formation of alkaline minerals (Lehmann et al., 2011; Panwar and Pawar, 2020). This alkaline shift can be beneficial in acidic soils, enhancing nutrient availability and microbial activity (Mukherjee et al., 2014; Shi et al., 2019). The increase in AP and AK at TEP4 suggests that high-temperature biochar enhances phosphorus and potassium availability. This could be attributed to the breakdown of organic phosphorus and the concentration of mineral-bound phosphorus in biochar at higher temperatures (Mukherjee and Zimmerman, 2013; Yuan and Xu, 2011). Similarly, potassium retention is improved due to biochar’s ability to adsorb and slowly release exchangeable K + ions, making them more available for plant uptake (Hossain et al., 2020; Laird et al., 2010). However, the lower ammonium nitrogen (NH₄^+^-N) content at TEP4 compared to TEP2 suggests that high-temperature biochar may promote ammonia volatilization, reducing its retention in the soil matrix (Enders et al., 2012; Liu Q. et al., 2019). In contrast, the increased nitrate nitrogen (NO_3_^−^-N) at TEP4 suggests an enhancement in nitrification, possibly due to improved soil aeration and microbial activity (Xu et al., 2014). These findings indicate that selecting an optimal pyrolysis temperature is crucial for balancing soil pH, nutrient retention, and microbial-driven nutrient cycling.

Seedling growth and biomass accumulation were significantly influenced by pyrolysis temperature, with TEP3 exhibiting the highest above-ground and total biomass. The positive effect of moderate-temperature biochar on plant growth is consistent with previous findings showing that biochar produced at 300–400 °C optimizes soil aeration, water-holding capacity, and nutrient retention, leading to enhanced plant performance (Lehmann and Joseph, 2015; Lehmann and Joseph, 2024). The increased availability of essential nutrients such as phosphorus and potassium in the TEP3 treatment likely contributed to improved root and shoot development, supporting higher biomass production (Agegnehu et al., 2016). Although TEP4 exhibited the tallest plants, total biomass did not follow the same trend, suggesting that extreme pyrolysis temperatures may negatively impact root growth and overall biomass accumulation. This could be due to reduced bioavailable carbon and other nutrient availability at higher temperatures, leading to lower microbial activity and nutrient mineralization (Song et al., 2021). These results suggest that biochar produced at moderate temperatures (~300 °C) provides the best balance between nutrient retention, soil microbial enhancement, and plant growth promotion.

Metagenomic sequencing revealed distinct shifts in soil microbial communities across pyrolysis temperature treatments. The increased relative abundance of *Pseudomonadota* in TEP2 suggests that lower-temperature biochar promotes microbial groups associated with organic matter decomposition and plant growth-promoting activities (Bhattacharyya et al., 2024). This result aligns with previous findings showing that biochar produced at lower temperatures retains more labile organic compounds, which serve as carbon sources for heterotrophic bacteria (de Oliveira Paiva et al., 2024). Conversely, the higher abundance of *Acidobacteriota* in TEP3 and TEP4 suggests that higher pyrolysis temperatures create conditions favoring oligotrophic, acid-tolerant microbes (Liang et al., 2019). These microbes thrive in soils with limited labile carbon availability, relying on stable organic compounds for energy metabolism. The increased presence of *Chloroflexota* and *Gemmatimonadota* across biochar treatments further highlights their role in nutrient cycling, particularly in nitrogen transformation and carbon mineralization (Liu X. et al., 2019). These findings suggest that biochar-induced microbial shifts are driven by changes in soil chemistry, carbon availability, and nutrient dynamics.

The observed increase in microbial richness (Chao, ACE, and Sobs indices) with higher pyrolysis temperatures indicates that biochar provides a structurally complex habitat that supports microbial colonization. However, the Shannon index was highest at TEP2, suggesting that moderate temperatures create a more balanced microbial community structure. This is consistent with studies showing that biochar influences microbial diversity by modifying soil chemical properties and providing microhabitats (Li et al., 2024). The significant differences in *β*-diversity across treatments, as revealed by NMDS analysis, further confirm that pyrolysis temperature plays a major role in shaping microbial community composition and functional potential.

Biochar-induced changes in microbial functional genes highlight the role of pyrolysis temperature in regulating soil metabolic processes. The enrichment of fatty acid degradation genes (ACADM acd) at lower temperatures (TEP2) suggests that biochar produced at lower temperatures retains more bioavailable organic compounds, stimulating microbial lipid metabolism (Yang et al., 2022). In contrast, the stabilization of TCA cycle genes (ACSS1_2 acs, ACO acnA) at TEP3 and TEP4 indicates that moderate-to-high temperatures enhance microbial respiration and energy metabolism, potentially due to the increased recalcitrance of biochar-derived carbon (Liu et al., 2017). For nitrogen metabolism, the upregulation of nitrate reduction genes (narG, narZ, nxrA) at TEP3 suggests that moderate pyrolysis temperatures enhance microbial denitrification, supporting nitrogen retention in the soil (Wang et al., 2023; Wang et al., 2016). The increased expression of nitrite reductase (nirK) at TEP4 further supports this, indicating that higher pyrolysis temperatures promote denitrification processes. However, the suppression of ammonia assimilation genes (GLUD1_2 gdhA) at TEP2 suggests that intermediate temperatures may limit microbial nitrogen retention, potentially affecting plant nutrient uptake. These results highlight the importance of pyrolysis temperature in shaping microbial-driven nitrogen transformations.

The divergent effects of carbon and nitrogen metabolism on microbial diversity underscore the intricate interactions between metabolic pathways and microbial community structure. The observed positive correlation between carbon metabolism-related genes and microbial diversity aligns with previous findings that highlight carbon’s fundamental role as both an energy source and structural component for microbial communities (Wu et al., 2024). A greater diversity of carbon substrates expands microbial metabolic pathways, enabling a broader range of microorganisms to coexist and fulfill niche-specific functions, ultimately fostering higher species diversity (Baquero et al., 2021). This is particularly relevant in biochar-amended soils, where the porous structure of biochar provides microhabitats and enhances carbon availability, thereby supporting a more complex microbial ecosystem (Li et al., 2020).

Conversely, the negative correlation observed between nitrogen metabolism-related genes and microbial diversity suggests that increased nitrogen-cycling activities may impose selective pressures that reduce overall species diversity. This phenomenon can be attributed to competitive exclusion, wherein specific nitrogen-transforming microorganisms dominate the ecosystem due to their specialized metabolic capabilities (Kuypers et al., 2018). The negative correlation observed between microbial diversity and nitrogen metabolism (*R*^2^ = 0.56) suggests a potential functional trade-off within the soil microbial community. While competitive exclusion may partly explain this pattern, the enrichment of specific functional phyla under different pyrolysis temperatures appears to play a key role. In particular, high-temperature biochar (TEP4) promoted the relative abundance of *Chloroflexota*, a phylum recognized for its metabolic versatility in degrading complex organic matter and participating in nitrogen transformations such as denitrification and nitrate reduction (Freches and Fradinho, 2024; Petriglieri et al., 2023). The dominance of *Chloroflexota* may therefore enhance nitrogen metabolism functions, even as overall microbial diversity decreases, reflecting a shift toward functional specialization. In contrast, Proteobacteria—which were abundant across treatments—are widely known for their roles in nitrogen fixation, nitrification, and denitrification (Huang et al., 2022; Zhao et al., 2023), and their activity may contribute to maintaining nitrogen turnover under biochar amendment. Similarly, Actinobacteria are involved in organic nitrogen decomposition and ammonification (Lin et al., 2022), further supporting nitrogen cycling processes. Together, these findings imply that high-temperature biochar does not simply increase microbial activity uniformly but rather restructures the community toward specific functional groups. This restructuring enhances nitrogen metabolism through the enrichment of key phyla while reducing community diversity, suggesting a trade-off between microbial diversity and functional efficiency in biochar-amended soils. High rates of nitrification and denitrification can lead to the preferential proliferation of ammonia-oxidizing bacteria and denitrifiers, outcompeting other microbial taxa and leading to reduced community evenness (Cong et al., 2015; Purswani and Llorente, 2021). Additionally, the accumulation of reactive nitrogen species, such as nitrate and nitrite, can create chemical stressors that inhibit the growth of sensitive microbial groups, further shaping community composition (Zhang et al., 2025).

These findings emphasize the necessity of adopting a holistic approach to studying microbial metabolic interactions, as biochar amendments not only alter soil chemistry but also drive significant shifts in microbial functional dynamics. The ability of biochar to enhance carbon substrate availability while simultaneously modifying nitrogen cycling pathways suggests that pyrolysis conditions and biochar properties should be carefully optimized to promote microbial diversity and ecosystem stability (Wang et al., 2023; Wang et al., 2016). By integrating metagenomic analyses with biogeochemical assessments, future research can further elucidate the mechanisms underlying microbial responses to biochar amendments and provide insights into sustainable soil management practices.

RDA analysis identified ammonium nitrogen (NH₄^+^-N) and acid phosphatase activity (APA) as key environmental factors driving microbial community structure. The strong correlation between pH and microbial diversity supports the idea that biochar-induced pH shifts significantly impact microbial ecology (Dong et al., 2022). Additionally, the influence of phenylalanine ammonia-lyase (PAL) and chlorophyll b (PCIIb) on microbial functions suggests that secondary metabolism and photosynthetic activity play crucial roles in microbial community structuring. This study underscores the importance of pyrolysis temperature in determining the effects of *Flammulina velutipes* residue biochar on soil microbial diversity, nutrient cycling, and plant growth. Our findings suggest that biochar produced at moderate temperatures (~300 °C) provides the best agronomic benefits by enhancing soil fertility, microbial functional potential, and seedling biomass accumulation. Future research should focus on long-term field studies to validate these effects and further explore the mechanisms underlying biochar-microbe-plant interactions in various soil environments. This study demonstrated that *Flammulina velutipes* residue biochar significantly affects soil properties, microbial communities, and seedling growth, with pyrolysis temperature being a key determinant of these effects. Higher temperatures (TEP4) increased soil pH and phosphorus availability, while lower temperatures (TEP2) enhanced ammonium nitrogen retention. The TEP3 treatment optimized seedling biomass accumulation and microbial functional diversity, indicating that moderate pyrolysis temperatures best balance soil nutrient dynamics and plant growth.

Metagenomic sequencing revealed that different microbial taxa responded distinctively to biochar treatments, with *Pseudomonadota* thriving in lower-temperature biochar, while *Acidobacteriota* and *Chloroflexota* were enriched in TEP3 and TEP4. Functional gene analysis further indicated that carbon and nitrogen metabolism pathways were temperature-dependent, with nitrate reduction peaking at TEP3 and nitrite reduction at TEP4. These findings highlight the role of pyrolysis temperature in shaping microbial functions critical for nutrient cycling.

Overall, this study provides strong evidence that biochar produced at 300 °C offers the best agronomic benefits by enhancing soil microbial activity, nutrient retention, and plant growth. These insights contribute to optimizing biochar production and application strategies for sustainable agriculture. Future research should explore long-term field applications and interactions with different soil types to validate these findings under diverse environmental conditions.

## 5 Conclusion

This study demonstrates that the pyrolysis temperature of *Flammulina velutipes* residue biochar significantly influences soil physicochemical properties, microbial community composition, and seedling growth. Different pyrolysis temperatures exhibited distinct ecological effects: (1) High-temperature biochar (400 °C, TEP4) significantly increased soil pH and phosphorus-potassium availability, while promoting nitrite reduction. However, its impact on seedling biomass was limited. (2) Low-temperature biochar (200 °C, TEP2) facilitated ammonium nitrogen retention and increased the abundance of *Pseudomonadota* but had a weaker effect on overall nutrient supply. (3) Moderate-temperature biochar (300 °C, TEP3) provided the optimal balance of nutrient retention, microbial diversity, and plant growth, enhancing nitrate reduction enzyme activity, carbon metabolism, and seedling biomass accumulation.

Functional gene analysis indicated that carbon and nitrogen metabolic pathways responded differently to pyrolysis temperature. TEP3 favored nitrate reduction and microbial respiration, whereas TEP4 was more associated with nitrite reduction. Regression analysis identified a positive correlation (*R*^2^ = 0.75) between microbial diversity and carbon metabolism-related genes, while nitrogen metabolism-related genes were negatively correlated with microbial diversity (*R*^2^ = 0.56), suggesting that shifts in nitrogen availability may influence microbial competition. Redundancy analysis further indicated that ammonium nitrogen and soil enzyme activity were key drivers shaping microbial community structure.

In summary, our results demonstrate that biochar produced from *Flammulina velutipes* residue at 300 °C optimizes soil conditions by improving pH, nutrient availability, and microbial functional responses, thereby providing the most favorable environment for seedling cultivation. Importantly, this temperature is not only biologically effective but also practically feasible. Compared with higher pyrolysis temperatures (>500 °C), low- to medium-temperature biochar production (≈300–400 °C) requires lower energy input, achieves higher yield, and retains more labile organic fractions that are beneficial for soil fertility, while still ensuring adequate stability (da Silva et al., 2025; Hu et al., 2022). Although high-temperature biochar can provide greater structural stability, its higher production costs and reduced yield limit large-scale application (Uddin et al., 2022). Therefore, considering both soil ecological benefits and production feasibility, pyrolysis at 300 °C represents a practical and sustainable strategy for the valorization of golden mushroom residues into soil amendments.

## Data Availability

The data presented in the study are deposited in the SRA repository, accession number PRJNA1329984.
