# Supplementary material for: Pyrolysis temperature shapes biochar-mediated soil microbial communities and carbon-nitrogen metabolism
Source: Front Microbiol. 2025 Sep 26;16:1657149. doi: 10.3389/fmicb.2025.1657149 (PMC12511098; doi:10.3389/fmicb.2025.1657149)
Supplement: Supplementary file 1 [file Data_Sheet_1.docx]

**Pyrolysis Temperature Shapes Biochar-Mediated Soil Microbial Communities and Carbon-Nitrogen Metabolism**

Guihong Ren^a,b^, Wentao Shi^a^, Wenwen Li^a^, Jinlong Wang ^a,b,^*, Chunjuan Wang^a,^*, Guiyun Zhao^a,b^

^a^College of Science, Beihua University, Jilin, 132013, China

^b^Traditional Chinese Medicine Biotechnology Innovation Center in Jilin Province, Beihua University, Jilin 132013, China

*Correspondence: Jinlong Wang, [wangjl755@163.com](mailto:wangjl755@163.com); Chunjuan Wang, wangcj1022@126.com

**
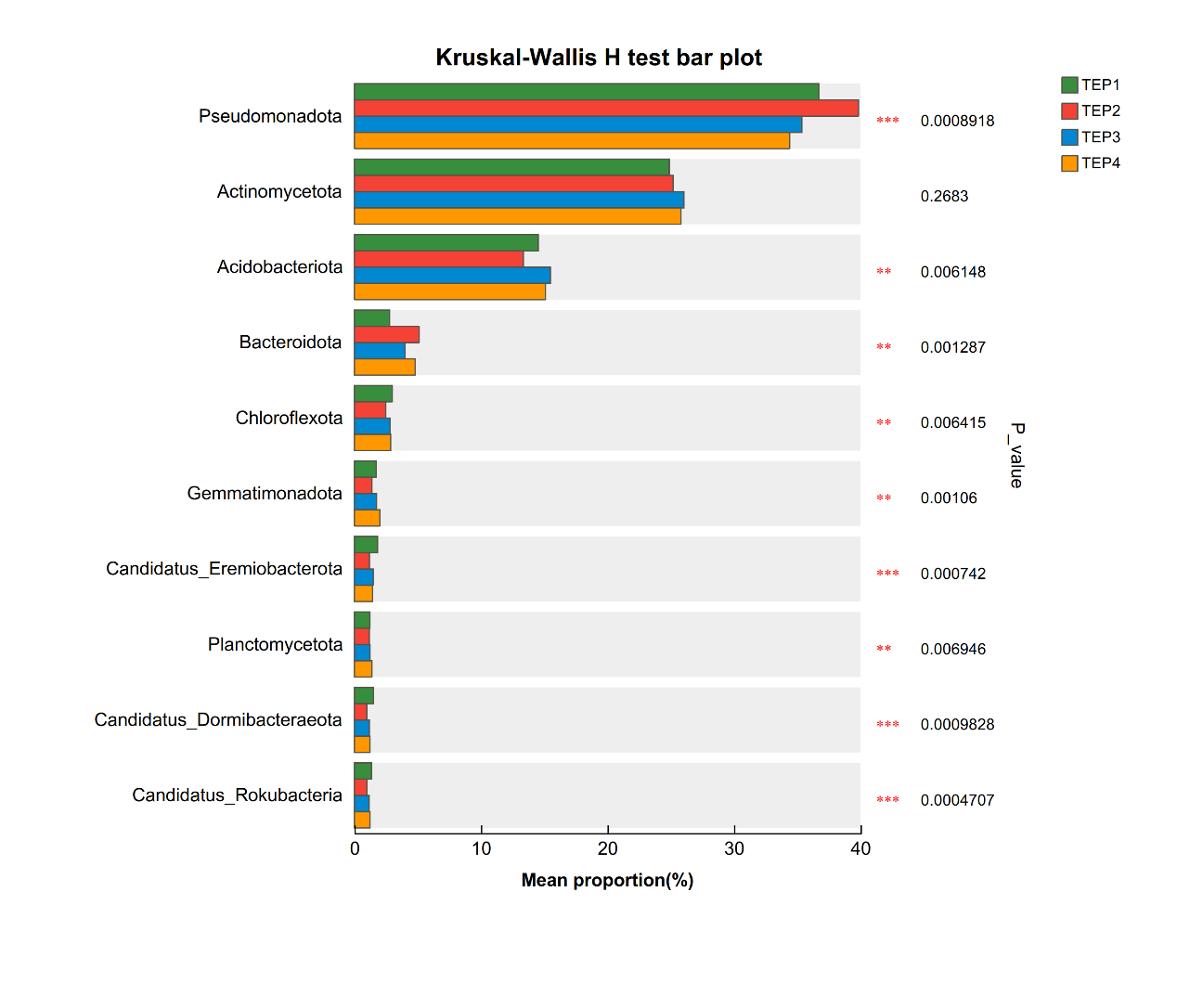
Figure S1.** Significant Differences in Soil Microbial Community Composition at the Phylum Level Across Pyrolysis Temperature Treatments


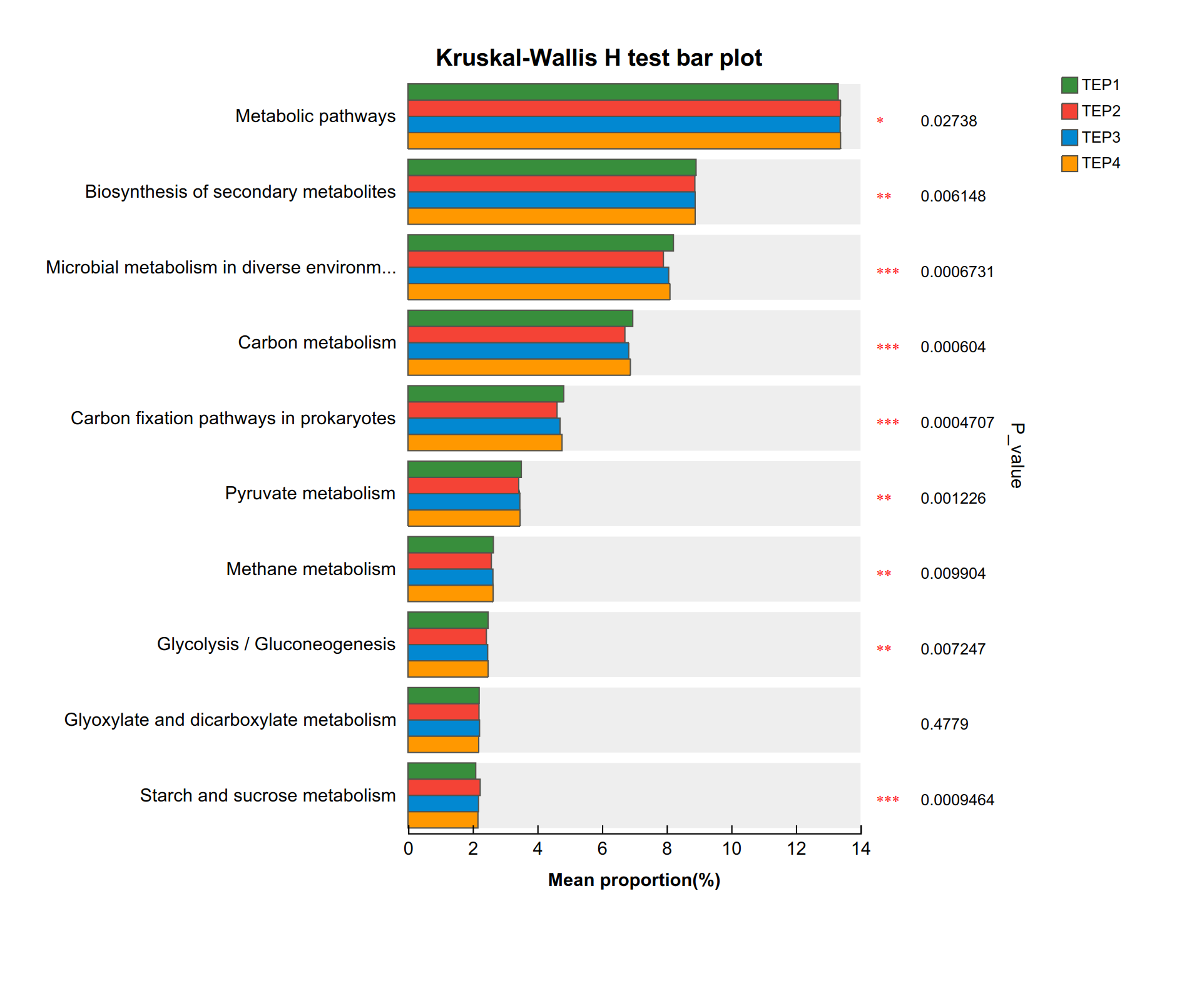


**Figure S2.** Significant Differences in Genes Related to Carbon Metabolism Across Pyrolysis Temperature Treatments


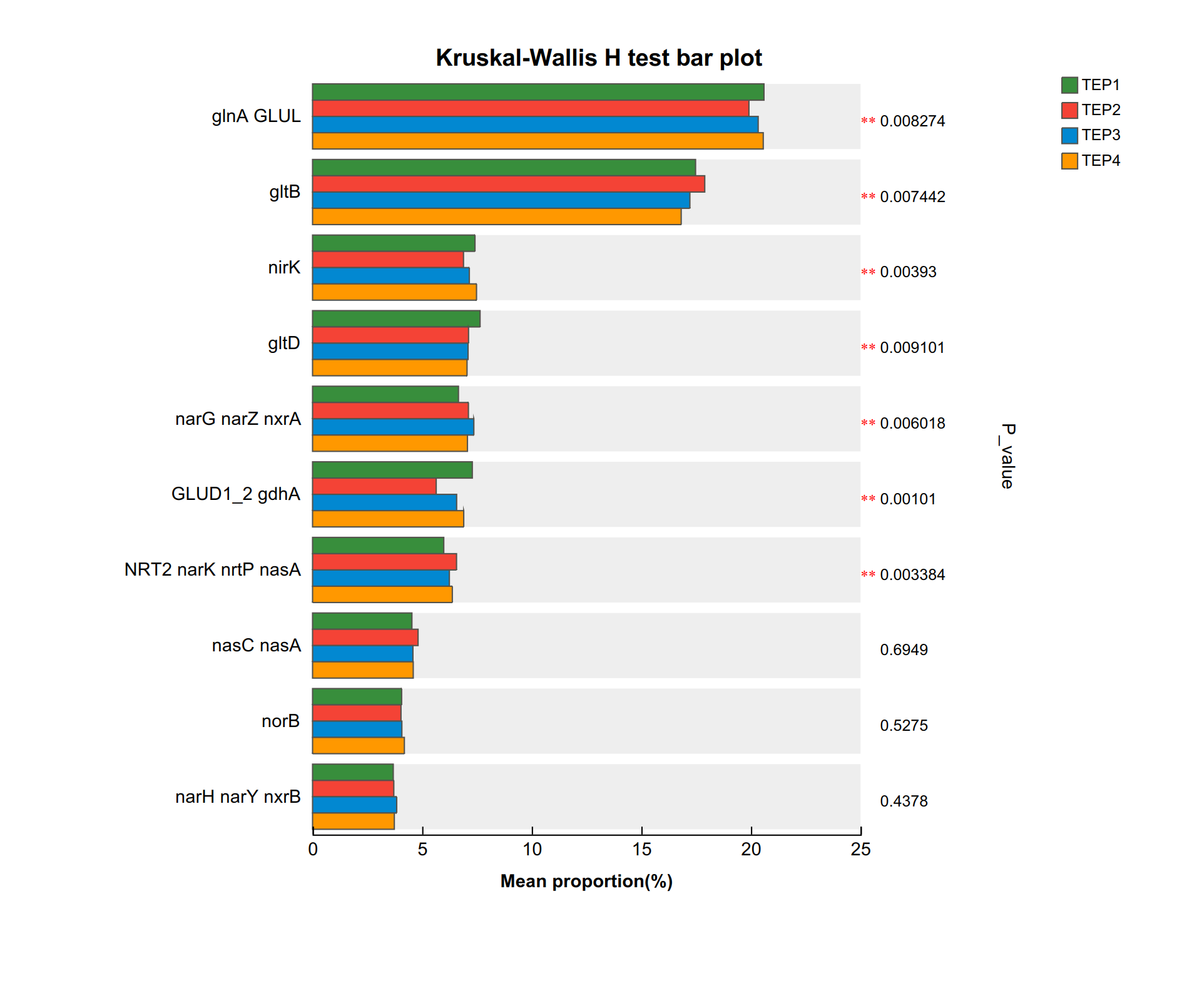


**Figure S3.** Significant Differences in Genes Related to Nitrogen Metabolism Across Pyrolysis Temperature Treatments
